# One-Piece Implants with Smooth Concave Neck to Enhance Soft Tissue Development and Preserve Marginal Bone Levels: A Retrospective Study with 1- to 6-Year Follow-Up

**DOI:** 10.1155/2018/2908484

**Published:** 2018-07-24

**Authors:** Jean-Pierre Axiotis, Paolo Nuzzolo, Carlo Barausse, Roberta Gasparro, Paolo Bucci, Roberto Pistilli, Gilberto Sammartino, Pietro Felice

**Affiliations:** ^1^“Centre of Implantology and Dental Aesthetics”, Région Stéphanoise, France; ^2^Department of Neurosciences, Reproductive Sciences and Dental Sciences, University Federico II of Naples, Italy; ^3^Department of Biomedical and Neuromotor Sciences, Unit of Periodontology and Implantology, University of Bologna, Bologna, Italy; ^4^Oral and Maxillofacial Unit, San Camillo Hospital, Rome, Italy

## Abstract

Novel one-piece implants with concave smooth neck have been introduced to promote the formation of a thick mucosal layer and preserve marginal bone. A retrospective study on 70 patients with 1- to 6-year follow-up was carried out. Cumulative survival rates were assessed. Variations of marginal bone level were measured on periapical radiographs as distance of the implant-abutment junction from the bone crest. Influence of different variables on treatment outcome was evaluated. Cumulative success rate after 6 years was 99.4 % at implant level and 98.6 % at patient level. Marginal bone level changed in a significant way over time. After 4 months, an increase of radiographic bone level of 0.173 ± 1.088 mm at implant level and 0.18 ± 1.019 mm at patient level was recorded. Mean marginal bone loss after 5 years was 0.573 ± 0.966 mm at implant level and 0.783 ± 1.213 mm at patient level. Age, sex, smoking habits, implant sites, implant lengths and diameters, prosthetic retentions, and timing of loading did not influence marginal bone remodeling in a statistically significant way. At 4-year follow-up partial restorations lost a mean of 0.96 mm of more marginal bone compared with single restorations. This difference was statistically significant.

## 1. Introduction

The maintenance of peri-implant marginal bone level is the key to long-term functional and esthetic outcome of implant-supported restorations. Together with the absence of pain, inflammation, mobility, and radiographic radiolucency between implant and bone, a marginal bone loss lower than 2 mm is a mandatory criterion of success [1].

Many factors have been advocated to explain marginal bone resorption around a healthy osseointegrated implant: the establishment of a biological width, the occlusal trauma, the gingival biotype, the surgical trauma, the micromovements of the abutment, retrieved cycles of connection and disconnection of the abutment, the bacterial colonization of the implant-abutment junction (IAJ), the distance of the IAJ from the bone crest, and the implant micro- and macrogeometry [2–6]. Still, the etiological factors underlying marginal bone loss have not been fully established [5, 7]. Implant neck morphology has been widely investigated in order to find designs that would promote bone ingrowth or limit bone loss and favour the creation of a steady mucosal seal [8].

Implant neck surface characteristics have also proven some relevance on the soft and hard tissues architecture [9]. The question whether a polished or a rough surface is more favourable for bone preservation is still debated [10–12]. More recently, one-piece implants have been introduced with a novel neck design, in which the transmucosal component has a narrower diameter than the implant body and a concave smooth surface meant promoting soft tissue creeping and the formation of a thick mucosal layer, which develops in a horizontal plane and, as such, is not created at the expenses of the underlying marginal bone. With respect to traditional flared implant necks, this new design provides more space for soft tissues ingrowth and organization.

Given the encouraging preclinical data, the aim of the present retrospective study was to analyse the long-term marginal bone preservation around 167 implants with a concave transmucosal design placed in 70 patients with 1 to 6 years of follow-up.

## 2. Materials and Methods

**Figure 1:**
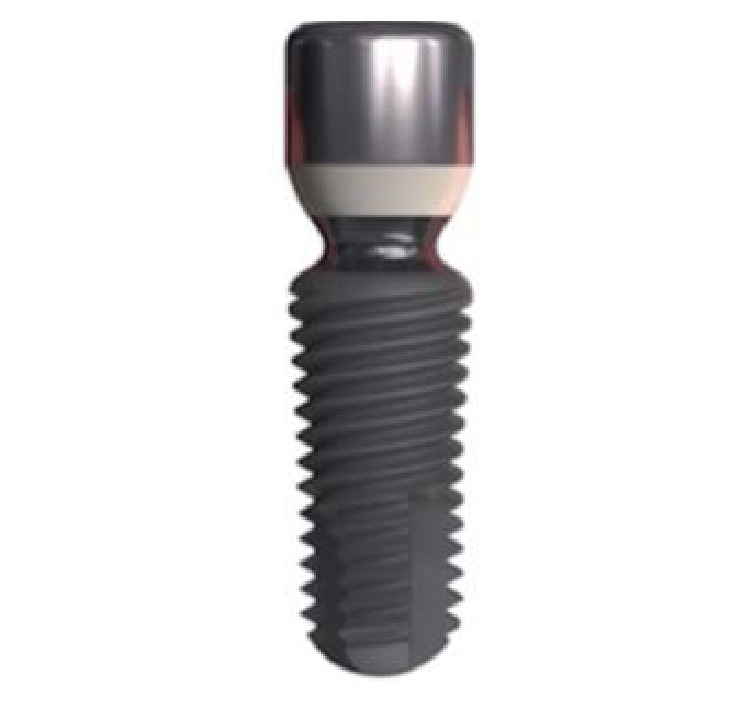
Twinkon implant with concave smooth transmucosal neck.

The investigation design was a retrospective study. Clinical and radiographic documentation of 70 patients that had been treated with the placement of a total of 167 commercially available sand blasted Ti-6Al-4V implants with concave smooth neck (Twinkon®, Global D, Brignais, France) was collected and analysed. This implant has a one-piece design with external conical connection, which is protected with a PEEK plastic ring. The concave transmucosal part is 1.5 mm high and 1.73 mm long (Figure 1). The horizontal inward mismatch between the implant body and the transmucosal component is 0.4 mm. The sandblasted surface (sprayed with corundum micropowder) extends to the apical portion (0.20 mm high) of the transmucosal neck. The coronal portion of the transmucosal neck is machined (1.3 mm high). The selection criteria for the cases were the availability of periapical radiographs at baseline and at follow-up/s, clinical information about sex, age, smoking habits, implant site/s, insertion torque (< or > 25N/cm), implant/s length/s and diameter/s, postextractive or delayed placement, single, partial, or full-arch restorations, screw-retained or cemented prostheses, months of healing before prosthetic load, and report of complications. Files were excluded if incomplete or shorter than one year of follow-up and if radiographic identification of the bone crest level was questionable. All the patients displayed good general health without systemic or local contraindications to oral surgery and did not suffer from active periodontitis at the time of implant placement. Patients received proper information about the surgical procedures, risks and alternative solutions, and signed an informed consent for the analysis and divulgation of their clinical information for scientific purposes. The principles outlined in the Declaration of Helsinki (64^th^ revision) on clinical research involving human subjects were adhered to.

### 2.1. Surgical and Prosthetic Procedures

Following proper clinical and radiographic evaluation, the patients underwent professionally delivered oral hygiene and, if required, scaling and root planning, prior to implant placement. Patients were given prophylactic antibiotic therapy with 2 g of amoxicillin plus clavulanic acid (or clindamycin 600 mg, if allergic to penicillin) 1 h before the intervention and postoperatively 1 g amoxicillin plus clavulanic acid twice a day, for 5 days, or 300 mg clindamycin twice a day, for 7 days. The surgical procedure was performed under local anaesthesia. After a full-thickness crestal incision, a mucoperiosteal flap was elevated, and implant tunnels were realized with drills of increasing diameter under generous sterile saline irrigation. All implants were placed in native bone. Implants were placed to a depth varying on the clinical situation: as a general rule, in case of a delayed positioning in a healed ridge, the implant shoulder was placed at a crestal level, while in postextractive sockets it was placed 1,5 mm subcrestally, i.e., with the coronal end of the concave neck at a crestal level, according to standard manufacturer's protocol. Flaps were carefully sutured with resorbable sutures. X-rays were taken after implant placement to verify the correct implant position.

Ibuprofen (600 mg) was prescribed to be taken as needed. A cold and soft diet was recommended for 2 weeks and oral hygiene instructions were given. Patients were instructed to rinse twice daily with 0.12% chlorhexidine digluconate for the first 2 weeks. Sutures were removed 7 days after the surgery.

Depending on surgical and prosthetic considerations, immediate, early, or delayed loading was chosen to rehabilitate the patients. Provisional resin restorations were delivered immediately after implant placement in the case of immediate loading protocols or few weeks after abutment connection in the cases of delayed loading protocols. Definitive metal-ceramic restorations were cemented or screwed 2 to 4 months after provisional delivery.

All the patients were scheduled in a maintenance program with clinical and radiographic evaluation and oral hygiene recalls every 3 to 6 months.

### 2.2. Measurements

The primary outcome was the marginal bone level (MBL), measured on periapical radiographs, as linear distance in mm from the implant-abutment junction (IAJ) of the most coronal radiographic bone-implant contact (rx-fBIC). This distance was calculated on the mesial and distal aspect of each implant and given a positive sign if the rx-fBIC was coronal to the IAJ and a negative sign if it was apical to the IAJ. The measurements were realized using the Osirix software (Pixmeo Sarl, Bernex, Switzerland). As radiographs were not taken with a previously standardized technique, the biometric evaluations were calibrated on each radiograph using the height of the concave neck of the implant as known dimension (1,5 mm). Measurements were made to the nearest 0,1 mm. Variations of MBL from baseline were calculated on radiographs taken after 4 months and 1, 2, 4, 5, and/or 6 years, depending on the availability of data, and expressed as means and standard deviations.

Subgroup analyses were carried out to assess the influence on the changes in MBL of these variables: sex, age, smoking habits, implant sites, insertion torque (< or > 25N/cm), implant length and diameter, postextractive or delayed placement, single, partial, or full-arch restoration, screw- retained or cemented prosthesis, and months of healing before prosthetic load.

The analysis was carried out at patient level and implant level.

Implant survival and success rate were assessed following the guidelines for studies on endosseous implants [13–15]: absence of pain, mobility, suppuration, mucosal redness and swelling, foreign body sensation, presence of plaque, and marginal bone loss. If all the parameters were satisfied and marginal bone loss was lesser than 1,5 mm in the first year of function and 0,2 mm for the following year, the case outcome was considered as success otherwise as survival. Rates were calculated as percentages in each time-frame considered.

### 2.3. Statistical Analysis

Descriptive statistics were used to summarize data: frequencies were used for nominal-level variables; means, standard deviations, and ranges were used for ordinal and continuous data. A log rank test was run to investigate differences in the implant and prosthetic survival distribution with respect to implant location (maxillary or mandibular arch, anterior or posterior site, and postextractive or not postextractive site), timing of loading (immediate or delayed), prosthesis type (single or partial), and implant-supported restoration type (screwed or cemented).

Marginal bone levels differences over time were investigated at site level (mesial and distal measurements), at implant level (mean between mesial and distal measurements), and at patient level (mean among the different implants of the same subject). At patient level a repeated measures ANOVA was used, whereas a repeated measures analysis including both fixed (time) and random effects (subject) was performed at site and implant level to account for the within-subject inner correlation.

Marginal bone levels changes between the different time points and baseline were calculated. They were compared with respect to implant location (maxillary or mandibular arch, anterior or posterior site, and postextractive or not postextractive site), timing of loading (immediate or delayed), prosthesis type (single or partial), and implant-supported restoration type (screwed or cemented) through t-tests at patient level and through nested ANOVAs at site and implant level (clustered data).

All statistical analyses were conducted using the Statistical Package for Social Sciences Software (SPSS Statistics Release 21, IBM, New York, USA). P < 0.05 was set as the level for statistical significance.

## 3. Results

**Figure 2:**
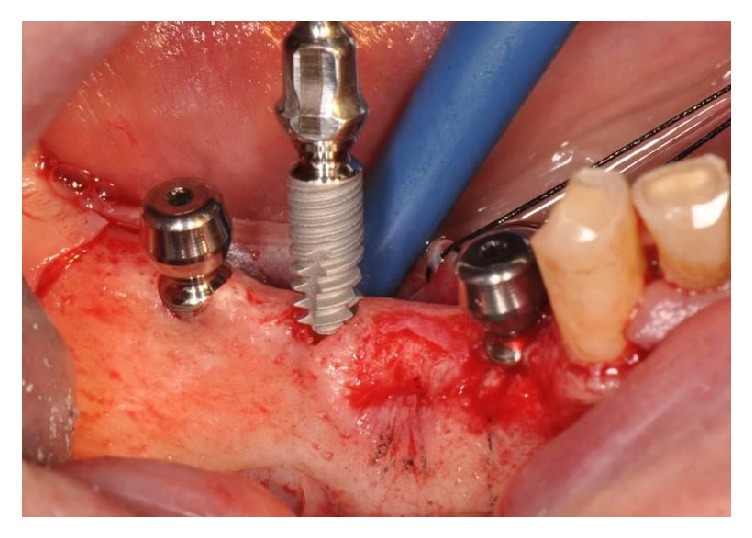
Implant placement in healed ridge for a partial restoration in posterior mandible. The smooth concave neck is left above the bony crest to allow soft tissues maturation.

Patient and intervention characteristics are summarized in Table 1. A total of 70 patients (45 females, 25 males; age range: 22 to 77 years, mean age at the beginning of the treatment: 56 years) were treated with 167 implants. The patients were treated between 2009 and 2012 by the same experienced surgeon. A small proportion of patients (17.1%) were smokers; half of the smokers were classified as heavy smokers (more than 10 cigarettes/day). The majority of the patients received 1 to 2 implants (65.8%), with a range from 1 to 7 implants per patient. The mandibular arch alone was the most often treated (72.9 % of the implants), and implants were placed in the posterior sectors of the upper and lower jaws in 82.2 % of the cases. Mean implant length and diameter were 9.74±1.66 and 4.25±0.8, respectively. A total of 33 postextractive implants were placed in 17 patients. All but five implants were placed with a torque > 25 Ncm. A total of 30 single and 41 partial prostheses were delivered, while no full-arch rehabilitation was realized. Regarding prosthetic retention, 50 restorations were cemented, while 15 were screw-retained; in 3 cases the information was not reported. A mean time of 3.58±2.32 months before prosthetic loading was calculated. Data from 4-month, 1-year, 2-year, 4-year, 5-year, and 6-year follow-up were collected when available. Due to the limited number of records, 2-year and 6-year follow-up data were included in the computation of survival and failure rates but excluded from other statistical analyses (Figure 2). Implant and prosthetic failures are summarized in Table 2.

Of all the implants placed, one was lost for peri-implantitis after 4 years (cumulative success rate: 99.4 % at implant level; 98.6 % at patient level). The failed implant had been placed in a healed ridge (not postextractive) and had been loaded after 3 months, supporting a partial prosthesis of four elements on four implants in the posterior mandible. No significant influence in treated arch, site (anterior or posterior), prosthetic rehabilitation (single or partial), timing of placement, and loading on the occurrence of implant failure was detected. Similarly variables as age, sex, smoking habit, implant length, and diameter did not influence these rates in a statistically significant way. Two minor prosthetic complications (screw loosening) were also recorded.

**Figure 3:**
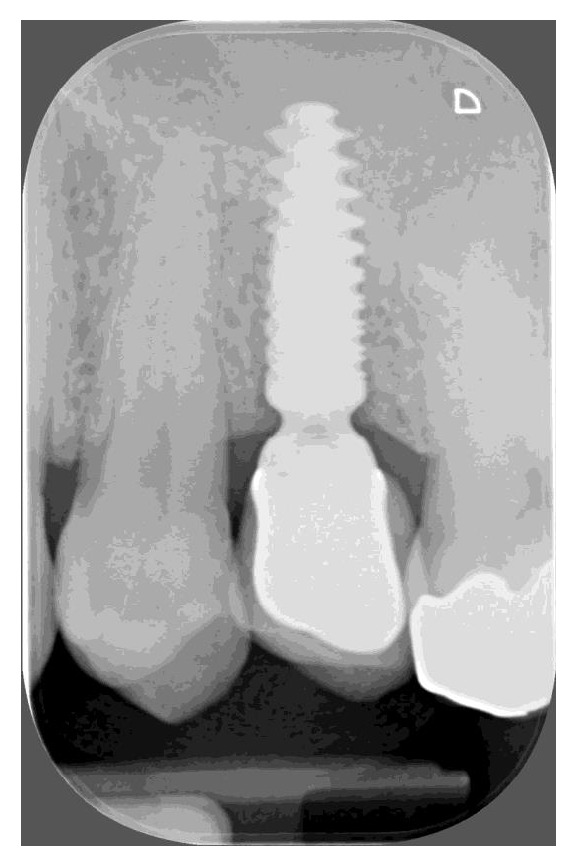
Radiographic 5-year follow-up of a single restoration. Some bone growth over the shoulder of the implant can be detected.

Mean peri-implant marginal bone level changes are shown in Table 3. MBL changed in a significant way over time, at site level, at implant level, and at patient level (p: 0.00, 0.00, and 0.002, respectively). After 4 months, a slight increase of radiographic level was recorded (mean value: 0.18 mm ± 1.019 at patient level) with respect to baseline, even though it did not reach statistical significance. At site level, mesial sites showed significant changes after 4 years and 5 years with respect to baseline and 4-month follow-up; distal sites showed significant changes after 1 year, 4 years, and 5 years with respect to baseline and after 4 years and 5 years with respect to 4-month follow-up. At implant level, significant changes were recorded after 1 year, 4 years, and 5 years with respect to baseline and after 4 years and 5 years with respect to 4-month follow-up. At patient level, significant changes were recorded at 5 years with respect to baseline and 4-month follow-up. Mean marginal bone loss after 5 years was 0.573 ± 0.966 mm at implant level and 0.783 ± 1.213 mm at patient level (Figure 3). The influence of different variables on marginal bone level changes was assessed (data not shown). No statistically significant differences in marginal bone level changes in relation to the arch treated and location in the arch (anterior or posterior) were detected. Prosthetic retention, screwed or cemented, had no significant influence on marginal bone remodeling, while a significant difference was found between single and partial restorations for changes after 4 years with respect to baseline at implant level and patient level (p value: 0.002, 0.003, and 0.003, respectively). Partial restorations were found to be subject to more bone resorption than single restorations (mean difference: 0,96 mm at patient level) after 4 years. The timing of loading had no significant influence on marginal bone remodeling, while the timing of implant placement was found to be determinant in a significant way only for distal sites after 4 years with respect to baseline: postextractive implants lost a mean of 1.003 mm more than not postextractive implants on the distal side (p= 0.03).

Variables as age, sex, smoking habits, implant length, and diameter did not influence marginal bone remodeling in a statistically significant way.

## 4. Discussion

The aim of this study was to assess the short- and long-term alterations of the hard tissues around one-piece implants with concave smooth neck. Marginal bone is the part of the peri-implant tissues at major risk of resorption. It has been shown that masticatory stresses are concentrated at this level, and bone loss can occur as a response to mechanical trauma [13, 16–18]. Furthermore, implant-abutment junction is located in this area, and it is the weakest part of the implant-restoration complex, both from a mechanical point of view and, mostly, from a biological standpoint. Irrespective of the kind of connection, a microgap between implant and abutment will always be present [14] and as a consequence bacterial microleakage will turn out into marginal bone loss [15, 19, 20]. Finally, even before prosthetic loading, when the implant is connected to a prosthetic or healing abutment, the soft tissues will always need a space to create a connective- epithelial seal around the transmucosal component. This biological width will be created at the expenses of the bony tissue if sufficient contact area is not provided [3].

The novel neck configuration analysed in this retrospective study has several advantages: the one-piece implant has a transmucosal neck, which brings the IAJ (and its microgap) in the soft tissues, away from marginal bone. The concave neck provides an increased space for soft tissues maturation and the establishment of a biological width. The incremented contact area provided by the concave design also provides major volume; it has been shown that, besides being shorter, the soft tissue seal around the implant is thicker [21]. The distance of 1,5 mm between the IAJ and the implant shoulder can be considered as a “safe distance” that prevents potentially harmful periodontal flora, which is known to extend apically from the epithelial junction to a maximum of 1,1 mm [22], from reaching the first bone to implant contact. Besides, the smooth surface of the neck prevents bacterial accumulation and the onset and progression of a peri-implantitis [23].

Three histologic studies showed promising results in terms of crestal bone preservation and soft tissues maturation with this concave transmucosal design. Bolle et al., in a histometric study on dogs, found evidence of some bone apposition on the implant shoulder during the healing: the marginal bone was at the level of the implant shoulder after 3 weeks and 0,18 mm above it after 18 weeks [21]. On the other hand, there have been controversial results about the dimensions of the biological width around this neck configuration: according to Huh and Bolle, this dimension is lower in the vertical plane compared to flared neck designs, while Kim et al. found no differences. In any case, it appears clear that a concave profile provides wider surface area given the same vertical dimension [21, 24, 25].

Monje et al. have shown that, together with factors as the quality of surgery, peri-implant bone thickness, and patient's habits, both the thickness of soft tissue and the location and characteristics of the IAJ are crucial for the preservation of peri-implant marginal bone [26]. The influence of implant geometry and surface on marginal bone remodeling has been stated in a meta-analysis by Laurell et al., in which a pooled mean bone loss varying from 0.24 mm to 0.75 mm was found, depending on the implant system [27].

Histologic evidence that implant design and surface are determinants in marginal bone level preservation has also been provided [28]. On the other side, Esposito et al. found no statistically significant difference in marginal bone preservation among different implant systems, even though the authors complained about a lack of well-designed RCTs for a proper meta-analysis [29].

In a retrospective multicenter radiographic evaluation of 596 dental implants, Cochran et al. found mean marginal bone loss of 2.84 ± 1.63 mm after 5 years. The authors also noted that 86% of the total mean bone loss had already occurred before prosthetic loading, so it should be ascribed to the healing pattern around the implants rather than to biomechanical factors [30].

In our study, mean peri-implant bone loss was of 0.57 mm after 5 years. More interestingly, peri-implant marginal bone did not resorb but rather overgrew on the implant shoulder to some extent after 4 months.

With this novel neck configuration, the formation of a mucosal attachment to the implant does not seem to happen at the expenses of the bony tissue; in fact, bony overgrowth seems to be promoted. The inevitable but acceptable bone loss over the years might still be related to biomechanical and/or microbiological factors, but the thickening of the soft tissues around the concave neck and the distance of the IAJ from the bone might have acted as protective factors that limited the extent of such resorption.

Even though the importance of soft tissue thickness for maintenance of peri-implant health has not been clearly defined yet [31], a review by Suárez-López Del Amo et al. demonstrated that marginal bone loss can be limited by thicker peri-implant mucosa [32]. Furthermore, implant dimensions, the arch treated and location (anterior or posterior), the kind of prosthetic span and retention, timing of placement and loading, and smoking habit did not influence success rate or marginal bone loss, with the exception of partial prostheses at 4-year follow-up and postextractive implants at 4-year follow-up.

This observation is in accordance with previous data in literature about the lack of influence on the implant therapy outcome of different implant sizes [33], single and partial rehabilitation [34], timing of restoration [35, 36], postextractive or delayed placement [37, 38], and cemented or screw-retained prosthesis [39]. While there is some evidence that smoking habits have a negative impact on the therapy outcomes [40, 41], we could find no difference between smokers and nonsmokers in our study. One possible explanation could be that a very limited number of patients enrolled in our study were heavy smokers.

In any case, this lack of interference of factors of different nature on the outcome of the therapy makes the implant evaluated in our study a viable solution for a vast range of different clinical situations.

## 5. Conclusions

Within the limits of this retrospective study, one-piece implants with concave smooth neck seem to ensure satisfactory success rates and long-term marginal bone preservation, irrespective of the implant dimensions, timing of placement and loading, and kind of rehabilitation. Further investigations, possibly in the form of well-designed RCTs, are needed to confirm the findings of this study.

## Figures and Tables

**Table 1:** Patient and intervention characteristics.

**# Patients**	70

**# Females**	45 (64.3%)

**Mean age at recruitment (range)**	55.64 (22-77)

Smokers	12 (17.1%)
smoking ≤ 10 cigarettes	6 (8.6%)
smoking > 10 cigarettes	6 (8.6%)

# **implants**	167

**# implants received by patient**	

# patients receiving 1 implant	23 (32.9%)

# patients receiving 2 implants	23 (32.9%)

# patients receiving 3 implants	10 (14.3%)

# patients receiving 4 implants	7 (10.0%)

# patients receiving 5 implants	4 (5.7%)

# patients receiving 6 implants	1 (1.4%)

# patients receiving 7 implants	2 (2.9%)

**Arch**	

# implants placed in Maxilla	17 (24.3%)

# implants placed in Mandible	51 (72.9%)

Both	2 (2.9%)

**Site**	

Anterior	30 (17.8%)

Posterior	139 (82.2%)

**# implants placed with ≤ 25 Ncm torque**	5 (3.0%)

**Mean implant length (mm)**	9.74±1.66

**Mean implant diameter (mm)**	4.25±0.8

**Post-extractive implants**	33

# patients receiving 1 post-extractive implant	8

# patients receiving 2 post-extractive implants	5

# patients receiving 3 post-extractive implants	1

# patients receiving 4 post-extractive implants	3

**Prosthesis**	

Single	29

Partial	40

Both	1

**Implant supported restorations**	

Screwed	15

Cemented	50

Not reported	5

**Months before loading**	3.58±2.32

**Table 2:** Implant and prosthetic failures.

	**4-month**	**1-year**	**2-year **	**4-year **	**5-year **	**6-year **
	**follow-up**	**follow-up**	**follow-up**	**follow-up**	**follow-up**	**follow-up**
**# implant failures**	n=87	n=9	n=1	n=100	n=59	n=1
	0 (0.0%)	0 (0.0%)	0 (0.0%)	1 (1.0%)	0 (0.0%)	0 (0.0%)
**# prosthetic failures**	n=42	n=6	n=1	n=55	n=31	n=1
	0 (0.0%)	0 (0.0%)	0 (0.0%)	1 (1.82%)	0 (0.0%)	0 (0.0%)

**Table 3:** Mean peri-implant marginal bone at baseline, at loading, at 4 months, and at 1, 4, and 5 years.

	**Baseline **	**4-month follow-up **	**1-year follow-up **	**4-year follow-up **	**5-year follow-up **	**Sig**	**Post**
	***Mean±SD***	***Mean±SD***	** *Mean±SD***	***Mean±SD***	***Mean±SD***		
**Implant ** **l** **e** **v** **e** **l** ^**∗**^	n=163	n=85	n=8	n=99	n=55		
	0.026 ± 0.775	0.173 ± 1.088	-0.386 ± 1.421	-0.383 ± 1.150	-0.573 ± 0.966	0.000	° # § ^*ϕ*^ ∧
**Patient ** **l** **e** **v** **e** **l** ^**∗∗**^	n=70	n=38	n=6	n=41	n=27		
	0.018 ± 0.734	0.182 ± 1.019	-0.295 ± 1.611	-0.184 ± 0.990	-0.783 ± 1.213	0.002	§ ∧

Data are presented as mean ± standard deviation. SD: standard deviation; Sig: significance; post: significant post hoc comparisons; °baseline vs 1 year; #baseline vs 4 years; §baseline vs 5 years; *∗*4 months vs 4 years; ∧4 months vs 5 years.

## Data Availability

Please contact professor Pietro Felice, email: pietro.felice@unibo.it.

## References

[1] Misch C. E., Perel M. L., Wang H.-L. (2008). Implant success, survival, and failure: the international congress of oral implantologists (ICOI) pisa consensus conference. *Implant Dentistry*.

[2] Sammartino G., Wang H. L., Citarella R., Lepore M., Marenzi G. (2013). Analysis of occlusal stresses transmitted to the inferior alveolar nerve by multiple threaded implants. *Journal of Periodontology*.

[3] Berglundh T., Lindhe J. (1996). Dimension of the periimplant mucosa. Biological width revisited. *Journal of Clinical Periodontology*.

[4] Ericsson I., Persson L. G., Berglundh T., Marinello C. P., Lindhe J., Klinge B. (1995). Different types of inflammatory reactions in peri-implant soft tissues. *Journal of Clinical Periodontology*.

[5] Hermann F., Lerner H., Palti A. (2007). Factors influencing the preservation of the periimplant marginal bone. *Implant Dentistry*.

[6] Hermann J. S., Schoolfield J. D., Nummikoski P. V., Buser D., Schenk R. K., Cochran D. L. (2001). Crestal Bone Changes Around Titanium Implants: A Methodologic Study Comparing Linear Radiographic with Histometric Measurements. *The International Journal of Oral & Maxillofacial Implants*.

[7] Prosper L., Redaelli S., Pasi M., Zarone F., Radaelli G., Gherlone E. F. (2009). A randomized prospective multicenter trial evaluating the platform-switching technique for the prevention of postrestorative crestal bone loss. *The International Journal of Oral & Maxillofacial Implants*.

[8] Yamanishi Y., Yamaguchi S., Imazato S., Nakano T., Yatani H. (2014). Effects of the implant design on peri-implant bone stress and abutment micromovement: Three-dimensional finite element analysis of original computer-aided design models. *Journal of Periodontology*.

[9] Schrotenboer J., Tsao Y.-P., Kinariwala V., Wang H.-L. (2008). Effect of microthreads and platform switching on crestal bone stress levels: A finite element analysis. *Journal of Periodontology*.

[10] Aloy-Prósper A., Maestre-Ferrín L., Peñarrocha-Oltra D., Peñarrocha-Diago M. (2011). Marginal bone loss in relation to the implant neck surface: An update. *Medicina Oral Patología Oral y Cirugía Bucal*.

[11] Esposito M., Felice P., Barausse C., Pistilli R., Grandi G., Simion M. (2015). Immediately loaded machined versus rough surface dental implants in edentulous jaws: One-year postloading results of a pilot randomised controlled trial. *European Journal of Oral Implantology*.

[12] Cosyn J., Sabzevar M. M., De Wilden P., De Rouck T. (2007). Two-piece implants with turned versus microtextured collars. *Journal of Periodontology*.

[13] Hansson S. (2000). Implant-abutment interface: biomechanical study of flat top versus conical.. *Clinical Implant Dentistry and Related Research*.

[14] Esposito M., Maghaireh H., Pistilli R. (2015). Dental implants with internal versus external connections: 1-year post-loading results from a pragmatic multicenter randomised controlled trial. *European Journal of Oral Implantology*.

[15] Piattelli A., Vrespa G., Petrone G., Iezzi G., Annibali S., Scarano A. (2003). Role of the microgap between implant and abutment: a retrospective histologic evaluation in monkeys. *Journal of Periodontology*.

[16] Pierrisnard L., Renouard F., Renault P., Barquins M. (2003). Influence of implant length and bicortical anchorage on implant stress distribution. *Clinical Implant Dentistry and Related Research*.

[17] Isidor F. (2006). Influence of forces on peri-implant bone. *Clinical Oral Implants Research*.

[18] Lum L. B. (1991). A biomechanical rationale for the use of short implants. *Journal of Oral Implantology*.

[19] Broggini N., McManus L. M., Hermann J. S. (2006). Peri-implant inflammation defined by the implant-abutment interface. *Journal of Dental Research*.

[20] Tsuge T., Hagiwara Y., Matsumura H. (2008). Marginal fit and microgaps of implant-abutment interface with internal anti-rotation configuration. *Dental Materials*.

[21] Bolle C., Gustin M.-P., Fau D., Exbrayat P., Boivin G., Grosgogeat B. (2015). Early periimplant tissue healing on 1-piece implants with a concave transmucosal design: A histomorphometric study in dogs. *Implant Dentistry*.

[22] Waerhaug J. (1976). Subgingival plaque and loss of attachment in periodontosis as observed in autopsy material. *Journal of Periodontology*.

[23] Sánchez-Siles M., Muñoz-Cámara D., Salazar-Sánchez N., Ballester-Ferrandis J. F., Camacho-Alonso F. (2016). Incidence of peri-implantitis and oral quality of life in patients rehabilitated with implants with different neck designs: a 10-year retrospective study. *Journal of Cranio-Maxillo-Facial Surgery*.

[24] Huh J.-B., Rheu G.-B., Kim Y.-S., Jeong C.-M., Lee J.-Y., Shin S.-W. (2014). Influence of Implant transmucosal design on early peri-implant tissue responses in beagle dogs. *Clinical Oral Implants Research*.

[25] Kim S., Oh K.-C., Han D.-H. (2010). Influence of transmucosal designs of three one-piece implant systems on early tissue responses: a histometric study in beagle dogs. *International Journal of Oral and Maxillofacial Implants*.

[26] Monje A., Suarez F., Galindo-Moreno P., García-Nogales A., Fu J.-H., Wang H.-L. (2014). A systematic review on marginal bone loss around short dental implants (<10 mm) for implant-supported fixed prostheses. *Clinical Oral Implants Research*.

[27] Laurell L., Lundgren D. (2011). Marginal bone level changes at dental implants after 5 years in function: a meta-analysis. *Clinical Implant Dentistry and Related Research*.

[28] Valderrama P., Bornstein M. M., Jones A. A., Wilson T. G., Higginbottom F. L., Cochran D. L. (2011). Effects of implant design on marginal bone changes around early loaded, chemically modified, sandblasted acid-etched-surfaced implants: A histologic analysis in dogs. *Journal of Periodontology*.

[29] Esposito M., Grusovin M. G., Coulthard P., Thomsen P., Worthington H. V. (2005). A 5-year follow-up comparative analysis of the efficacy of various osseointegrated dental implant systems: A systematic review of randomized controlled clinical trials. *The International Journal of Oral & Maxillofacial Implants*.

[30] Cochran D. L., Nummikoski P. V., Schoolfield J. D., Jones A. A., Oates T. W. (2009). A prospective multicenter 5-year radiographic evaluation of crestal bone levels over time in 596 dental implants placed in 192 patients. *Journal of Periodontology*.

[31] Martin W., Lewis E., Nicol A. (2009). Local risk factors for implant therapy. *International Journal of Oral and Maxillofacial Implants*.

[32] Del Amo F. S.-L., Lin G.-H., Monje A., Galindo-Moreno P., Wang H.-L. (2016). Influence of soft tissue thickness on peri-implant marginal bone loss: A systematic review and meta-analysis. *Journal of Periodontology*.

[33] Sotto-Maior B. S., Mercuri E. G. F., Senna P. M., Assis N. M. S. P., Francischone C. E., Del Bel Cury A. A. (2016). Evaluation of bone remodeling around single dental implants of different lengths: a mechanobiological numerical simulation and validation using clinical data. *Computer Methods in Biomechanics and Biomedical Engineering*.

[34] Firme C. T., Vettore M. V., Melo M., Vidigal G. M. (2014). Peri-implant bone loss around single and multiple prostheses: systematic review and meta-analysis. *The International Journal of Oral & Maxillofacial Implants*.

[35] Esposito M., Grusovin M. G., Willings M., Coulthard P., Worthington H. V. (2007). The effectiveness of immediate, early, and conventional loading of dental implants: a cochrane systematic review of randomized controlled clinical trials. *The International Journal of Oral & Maxillofacial Implants*.

[36] Suarez F., Chan H.-L., Monje A., Galindo-Moreno P., Wang H.-L. (2013). Effect of the timing of restoration on implant marginal bone loss: A systematic review. *Journal of Periodontology*.

[37] Chrcanovic B. R., Albrektsson T., Wennerberg A. (2015). Dental implants inserted in fresh extraction sockets versus healed sites: a systematic review and meta-analysis. *Journal of Dentistry*.

[38] Felice P., Soardi E., Piattelli M., Pistilli R., Jacotti M., Esposito M. (2011). Immediate non-occlusal loading of immediate post-extractive versus delayed placement of single implants in preserved sockets of the anterior maxilla: 4-month post-loading results from a pragmatic multicentre randomised controlled trial. *European Journal of Oral Implantology*.

[39] De Brandão M. L., Vettore M. V., Vidigal Júnior G. M. (2013). Peri-implant bone loss in cement- and screw-retained prostheses: Systematic review and meta-analysis. *Journal of Clinical Periodontology*.

[40] Moraschini V., Barboza E. D. P. (2016). Success of dental implants in smokers and non-smokers: a systematic review and meta-analysis. *International Journal of Oral and Maxillofacial Surgery*.

[41] Sayardoust S., Gröndahl K., Johansson E., Thomsen P., Slotte C. (2013). Implant survival and marginal bone loss at turned and oxidized implants in periodontitis-susceptible smokers and never-smokers: A retrospective, clinical, radiographic case-control study. *Journal of Periodontology*.

